# CCL3 Enhances Antitumor Immune Priming in the Lymph Node *via* IFNγ with Dependency on Natural Killer Cells

**DOI:** 10.3389/fimmu.2017.01390

**Published:** 2017-10-23

**Authors:** Frederick Allen, Peter Rauhe, David Askew, Alexander A. Tong, Joseph Nthale, Saada Eid, Jay T. Myers, Caryn Tong, Alex Y. Huang

**Affiliations:** ^1^Department of Pathology, Case Western Reserve University School of Medicine, Cleveland, OH, United States; ^2^Department of Pediatrics, Case Western Reserve University School of Medicine, Cleveland, OH, United States; ^3^Angie Fowler AYA Cancer Institute, UH Rainbow Babies & Children’s Hospital, Cleveland, OH, United States; ^4^Case Comprehensive Cancer Center, Cleveland, OH, United States

**Keywords:** CCL3, natural killer cells, CD103^+^ dendritic cells, lymphocytes, interferon-gamma, lymph node

## Abstract

Lymph node (LN) plays a critical role in tumor cell survival outside of the primary tumor sites and dictates overall clinical response in many tumor types (1, 2). Previously, we and others have demonstrated that CCL3 plays an essential role in orchestrating T cell—antigen-presenting cell (APC) encounters in the draining LN following vaccination, and such interactions enhance the magnitude of the memory T cell pool (3–5). In the current study, we investigate the cellular responses in the tumor-draining lymph nodes (TDLNs) of a CCL3-secreting CT26 colon tumor (L3TU) as compared to wild-type tumor (WTTU) during the priming phase of an antitumor response (≤10 days). In comparison to WTTU, inoculation of L3TU resulted in suppressed tumor growth, a phenomenon that is accompanied by altered *in vivo* inflammatory responses on several fronts. Autologous tumor-derived CCL3 (aCCL3) secretion by L3TU bolstered the recruitment of T- and B-lymphocytes, tissue-migratory CD103^+^ dendritic cells (DCs), and CD49b^+^ natural killer (NK) cells, resulting in significant increases in the differentiation and activation of multiple Interferon-gamma (IFNγ)-producing leukocytes in the TDLN. During this early phase of immune priming, NK cells constitute the major producers of IFNγ in the TDLN. CCL3 also enhances CD8+ T cell proliferation and differentiation by augmenting DC capacity to drive T cell activation in the TDLN. Our results revealed that CCL3-dependent IFNγ production and CCL3-induced DC maturation drive the priming of effective antitumor immunity in the TDLN.

## Introduction

Immunogenic tumors are capable of triggering robust antitumor immune responses. However, intrinsic and extrinsic factors, such as tumor cell growth rates and immune-suppressing factors, protect tumors from effective immune elimination (6). The balance between immune-mediated tumor suppression and evasion depends on many factors, including the timing, robustness, and types of responding immune cells within specific contexts of the local tissue microenvironments. Secondary lymphoid organs such as lymph nodes (LNs) play an important role in the development of these mediating factors. LNs are specialized sentinel stations designed to promote timely cellular interactions, disseminate antigenic information, and formulate adaptive responses that help to maintain tissue homeostasis. The importance of LNs in the spread of cancer is evident in the clinical environment where studies have reported that 80% of all tumor metastasis occur though LNs and that LN metastasis correlates negatively with clinical outcomes (7). Despite the important role LNs play in tumor progression, many of the current mechanistic insights of how immune cells respond in a tumor microenvironment (TME) come primarily from interrogations of cellular events that occur in the primary tumor sites. Studying immune interactions in the tumor draining lymph node (TDLN), not just in the primary tumor site, can provide important clues to local and global immune responses toward disseminating tumors (8, 9).

Chemokine-based immunotherapy has been studied as a means to modulate and bolster the development of antitumor immunity (10, 11). Chemokines are cytokines that function primarily as chemoattractants and help maintain specific immune microenvironments by coordinating various immune cell–cell interactions in a specific spatio-temporal manner. For example, we and others have demonstrated a crucial role for CCL3 and CCL4 in maximizing chance encounters between naïve CCR5^+^ CD8^+^ T cells and dendritic cells (DCs) that undergo productive interactions with antigen (Ag)-specific CD4^+^ or CD8^+^ T cells in the LN draining vaccine sites (3–5). Furthermore, CCL3 has been shown to be required for maximal helper CD4^+^ T cell-dependent memory CD8^+^ T cell generation in the DLN during the initial T cell priming phase (3). In the current study, we examined whether the chemoattractant and cytokine functions of CCL3 in the TDLN could be leveraged to improve immune responses to a murine colon cancer model by engineering tumors to secrete CCL3.

The cognate receptors for CCL3 include CCR5 and CCR1 in both mice and humans, and CCR3 in humans alone (12). Despite the differences of CCL3 isoforms and receptor binding between mice and humans, both species-specific CCL3 isoforms have been shown to eliminate tumors in mouse models (13). We hypothesize that the continuous presence of autologous tumor-derived CCL3 (aCCL3) in the TME will lead to the generation of a greater antitumor cellular response in the TDLN. Here, we employed CT26, a highly immunogenic murine colon tumor derived from BALB/c mice and expresses the immune-dominant Ag, AH-1, presented on the H2-L^d^ haplotype to CD8^+^ T cells (14, 15). We transfected wild-type CT26 tumors (WTTU) to stably secrete CCL3 (L3TU) and examine early cellular immigration of leukocytes to the TDLN. We show that CCL3 helps to maximize inflammatory responses in the TDLN in two parallel steps. First, we show that CCL3 increases the chance encounters of Ag-specific T cells with professional Ag-presenting cells (pAPCs) though the enhanced recruitment of CD103^+^ CD11c^+^ DCs (CD103+ DCs) and T cells in the TDLN. Second, CCL3 simultaneously enhances the global pool of interferon-gamma (IFNγ) in TDLN primarily through the mobilization of IFNγ-producing natural killer (NK) cells. Furthermore, we show that DCs exposed to recombinant CCL3 (rCCL3) exhibit enhanced Ag-presentation capacity to drive greater CD8^+^ T cell proliferation *in vitro*, thus demonstrating an alternative biological function of CCL3 from its classic cellular recruitment function to influence DC maturation.

## Materials and Methods

### Mice

Mice were purchased from the Jackson Laboratory (Bar Harbor, ME, USA) or Taconic. We used 8- to 12-week-old male and female wild-type BALB/c, C57BL/6J, or OT-I TCR transgenic mice on the RAG-1 knockout background. All animals were housed and handled according to National Institutes of Health institutional guidelines under an approved protocol by Case Western Reserve University Institutional Animal Care and Use Committee (No. 2012-0126 and 2015-0118).

### Tumor and LN Measurements

For tumor measurements, mice were injected s.c. with 1 × 10^6^ of each tumor construct alone or mixed at 1:1 ratio (5 × 10^5^ each) in the left flank, and tumors were measured twice weekly. Tumor growth was measured using electronic calipers. The formula, V = π × D × d2, was used to calculate tumor volumes (16), where “D” is the largest diameter and “d” is the smallest diameter. Mice were sacrificed between days 21 and 50, depending on the experimental setup.

For gross LN measurements, mice received s.c. footpad injections of tumor cells (1 × 10^6^), and the popliteal TDLN and non-draining lymph node (NDLN) were removed on day 5 or 7. High-resolution pictures were taken of TDLN and NDLN from each group (NI, WTTU, L3TU, WTTU + αAsialo-GM1, or L3TU + αAsialo-GM1) prior to FACS analysis. The large and small diameters of the LNs were measured in Adobe Illustrator software. The formula, V = π × D × d2, was used to calculate LN volumes (16). In order to normalize measurements from different photo sessions, each experiment was divided by the average of the WTTU group within the same experiment, and the relative fold-change was calculated.

### Flow Cytometry Analysis

Antibodies were purchased from eBioscience, BD Pharmingen, or BioLegend and are as follows: rat anti-mouse CD4 FITC and PE (GK1.5), APC (RM4-5); CD8a FITC, PE, and APC (53-6.7); CD19 FITC, PE, and APC (1D3); CD11c FITC, PE, and APC (N418); CD49b PE and APC (DX5); CD3 PE and APC (145-2C11); CD103 FITC (2E7); PD-L1 PE and APC (10F.9G2); CD69 PE and APC (H1.2F3); and IFNγ APC (XMG1.2). Mice received s.c. footpad injections of either tumor constructs and the popliteal TDLN and NDLNs were removed 1, 3, 5, 7, or 10 days later. LNs were made into single-cell suspensions with ice-cold FACS buffer (0.5% FBS and 0.5% EDTA in 1× sterile PBS). For surface staining, unlabeled rat anti-mouse blocking Fc antibody was applied for 30 min on ice followed by primary antibody staining for 30 min on ice and protected from light. Cell viability test was conducted using 7-AAD (Biolegend), which was added 20 min into the primary staining. The samples were then washed twice with ice-cold FACS buffer and analyzed on an Accuri C6 flow cytometer. For intracellular IFNγ staining, cells were plated with 1 μl/ml of GlogiStop (eBioscience) for 6-h on a 96 well plate pre-coated with unlabeled anti-CD3 at 1μg/ml for a total of 90 min at 37°C prior to membrane permeabilization with Cytofix/Cytoperm (BD Biosciences), followed by staining with anti-IFNγ antibody. Analysis was performed using Accuri C6 and FlowJo software.

### NK Depletion and CCL3 Blocking

For NK cell deletion, mice received intraperitoneal (I.P.) injections of 50 μg of αAsialo-GM1 (Poly21460) in 100 μl of 1× PBS. One day later, mice received s.c. footpad injections of 1 × 10^6^ tumor cells. 5 days later, the popliteal TDLN and NDLN were removed for FACS analysis. For CCL3 neutralization, mice received I.P. injections of anti-CCL3 antibodies (50 μg/mouse; R&D System) concurrently with 1 × 10^6^ WTTU or L3TU. Anti-CCL3 antibody injection was repeated again 48 h later. The popliteal TDLN and NDLN were removed 5 days later for FACS analysis.

### Enzyme-Linked Immunosorbent Assay (ELISA)

WTTU or L3TU were incubated for 24 h at 37°C in 95/5% O_2_/CO_2_ in 1 ml of complete media (RPMI 1640 with 10% fetal bovine serum, 1% HEPES, 1% non-essential amino acids, and 1% penicillin/streptomycin). The spent media from *in vitro* cultures or serum samples obtained from tumor-bearing mice were quantified for CCL3 protein contents by ELISA in accordance to the manufacture’s protocol (R&D systems, MMA00).

### CT26 Transfection

CT26 tumor cells were stably transfected with a PCDNA3.1 plasmid vector that contains the mouse CCL3 cDNA and maintained under Hygromycin (150 μg/ml) selection.

### OT-I Proliferation Assay

Bone marrows from C57BL6 mice were isolated and bone marrow-derived DCs (BMDCs) were generated in complete media at 3 × 10^6^ cells/3 ml/well in 6-well tissue culture plates supplemented with 15 ng/ml of granulocyte-macrophage colony-stimulating factor (GM-CSF) and 10 ng/ml interleukin-4 (IL-4) on days 0, 3, and 5. On day 3, the medium was removed and fresh media plus cytokines were added at 3 ml/well. On day 5, the cultures were replaced with fresh media plus cytokines. On day 7, non-adherent immature BMDCs were collected, washed with complete media and plated in a 6-well tissue culture plate with or without 100 ng/ml of CCL3 for 24 h. BMDCs were then collected and pulsed at 37°C in 95/5% O_2_/CO_2_ with various doses of SIINFEKL-peptide for 1 h, washed and cultured in triplicates with 100,000 CFSE-labeled (1 μM) naïve OT-I cells (at DC-to-OT-I ratio of 1:5) at 37°C in 95/5% O_2_/CO_2_ for 72 h. The percent of CFSE-dilution peaks, relative to non-pulsed and cultured BMDCs and OT-I cells, was calculated using FACS.

### Quantitative RT-PCR Analysis

Total LN mRNA was isolated using TRIzol reagent in accordance with the manufacturer’s protocol (Gibco BRL, Carlsbad, CA, USA) and purified using an Illustra™ RNAspin Mini Kit (GE Healthcare Life Sciences). RNA quality was assessed by spectrophotometer absorption at 260/280 nm using the NanoDrop2000 spectrophotometer. RNA was converted to cDNA using EasyScript™ Reverse Transcriptase protocol consisting of 200 U/μl Moloney murine leukemia virus reverse transcriptase incubated for 60-min at 42°C in the presence of 50 mM Tris-HCl (pH 8.3), 100 mM NaCl, 0.1 mM EDTA, 5 mM DTT, 0.1% Triton X-100, 50% (v/v) glycerol, 10 μM of oligo (dT), 10 mM 29-deoxynucleoside 59-triphosphate, and 40 U/μl recombinant RNase inhibitor (Lamda BIOTECH, St. Louis, MO, USA). cDNA was amplified in the presence of FAM-labeled gene-specific primers and Bullseye EvaGreen qPCR Mastermix (MIDSCI™; Saint Louis, MO, USA) in a 96 well microtiter plate using the ABI Prism 7300 sequence detection system (Applied Biosystems). Each PCR was performed in triplicate and compared to WTTU. Relative levels of mRNA were determined using the cycle threshold (C_t_). The gene expression was standardized according to cytochrome-*c* (Cy*C*) expression within the TDLN. In order to compare the C_t_ values between target genes, we normalized each C_t_ to the average of the WTTU C_t_ using the following equation: 2^^ − (Target gene − CyC − target normalizer)^.

### Statistical Analysis

**Figure 1:**
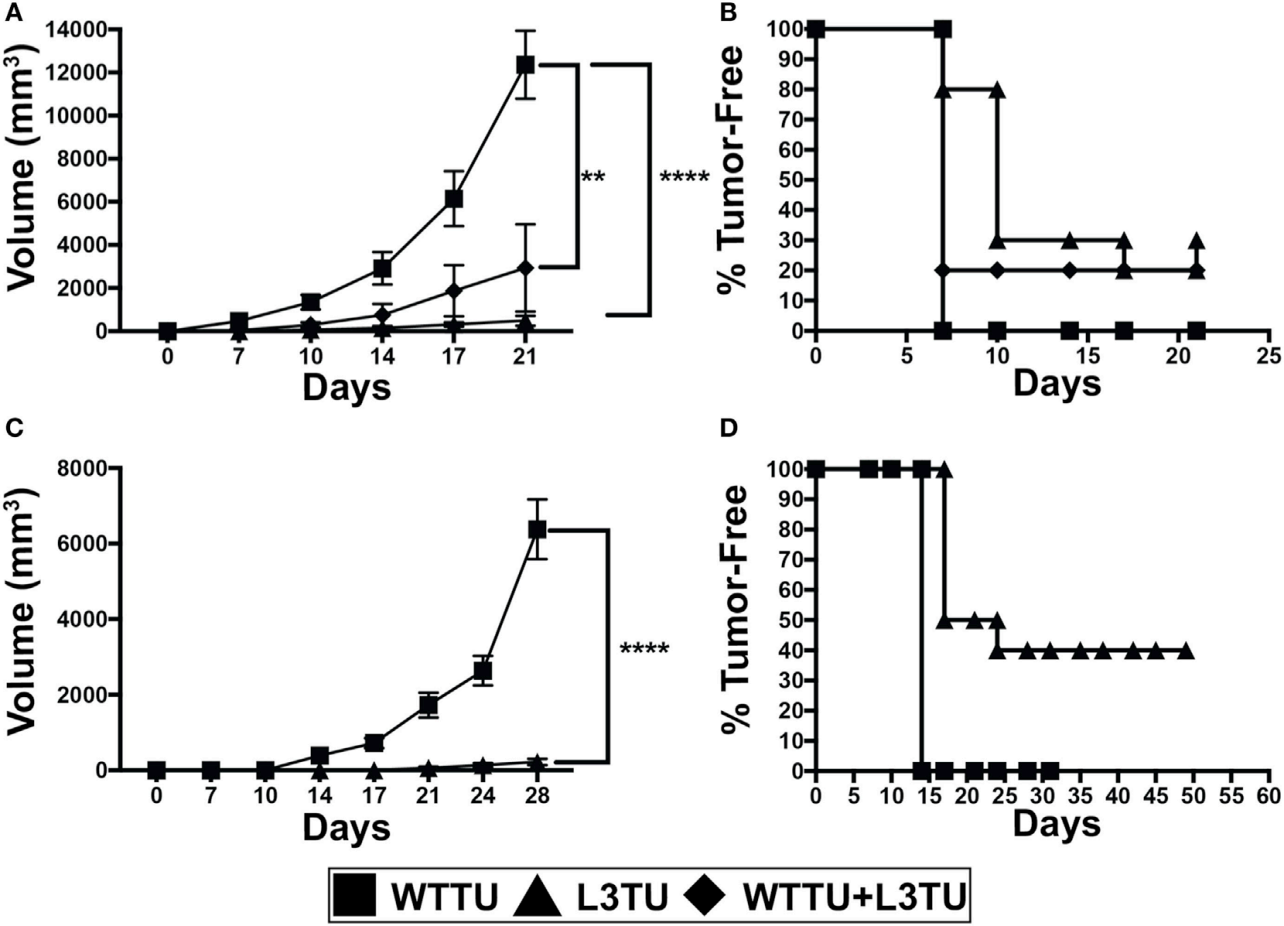
Autologous or recombinant CCL3 slows tumor growth and promotes tumor rejection. **(A)** Tumor growth kinetics of 1 × 10^6^ tumor cells injected s.c. of WTTU (*n* = 10), L3TU (*n* = 10), or WTTU + L3TU mixed at a ratio of 1:1 (*n* = 5). Experiments were repeated 3 times. **(B)** Kaplan–Meier graph showing overall tumor incidence of A. **(C)** Tumor growth kinetics of WTTU (*n* = 10) and L3TU (*n* = 10) after s.c. injections of 5 × 10^5^ tumor cells. **(D)** Kaplan–Meier graph showing overall tumor incidence of C. Not significant (ns), *p* > 0.05; *p = 0.01 to 0.05; **p = 0.001 to 0.01; ***p = 0.0001 to 0.001; ****p < 0.0001.

**Figure 2:**
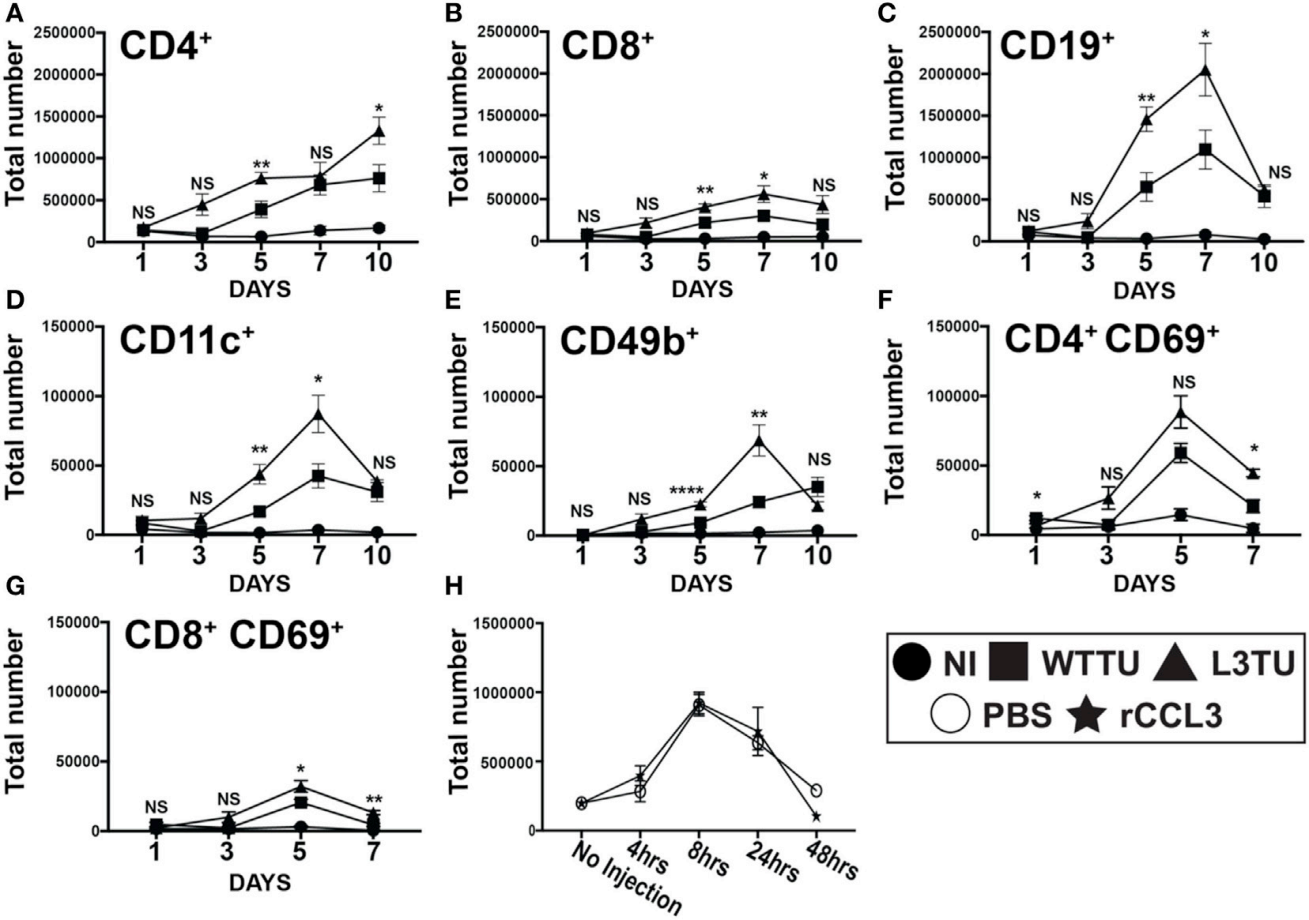
Autologous tumor-derived CCL3 (aCCL3) expression augments T cell activation by enhancing leukocyte migration to the tumor-draining lymph nodes (TDLNs). **(A–E)** Cellular accumulations of specific immune subtypes in the TDLN are shown over the course of 10 days following tumor inoculation. **(F,G)** Upregulation of the early activation marker, CD69, on T cells over the first 7 days following tumor inoculation. **(H)** Total cellularity changes in the popliteal lymph node of naive mice in the first 48 h following a footpad injection of recombinant CCL3 (rCCL3; 100 ng/mouse). For **(A–G)**, *n* = 3–7 mice for each day. Experiments were repeated two to three times for each data point. H represents two biological repeats with *n* = 2 mice in each time point. Not significant (ns), *p* > 0.05; *p = 0.01–0.05; **p = 0.001–0.01; ***p = 0.0001–0.001; ****p < 0.0001.

Significance analyses were performed using the standard *t*-test. Figures 1 and 2 were displayed as SEM for ease of visualization. SDs were shown for other figures.

## Results

### aCCL3 Suppresses Tumor Growth and Promotes Tumor Rejection

We first constructed L3TU by stably transfecting WTTU CT26 with plasmids containing murine CCL3. No significant differences in the growth kinetics between WTTU and L3TU were observed *in vitro* (Figure S1A in Supplementary Material). ELISA analysis revealed that L3TU produced ~350 pg/ml of CCL3 per 1 × 10^6^ tumor cells *in vitro* over 24 h, whereas WTTU failed to secrete any detectable CCL3 (Figure S1B in Supplementary Material). Serum obtained from mice 7 days after 1 × 10^6^ L3TU inoculation contained ~150 pg/ml CCL3, while serum from non-injected (NI) and WTTU-injected mice had negligible (< 8 pg/ml) CCL3 (Figure S1C in Supplementary Material). Both L3TU and WTTU expressed similar surface programmed death-ligand 1 (PD-L1) at baseline and following IFNγ stimulation in vitro (Figures S1D,E in Supplementary Material). Next, we measured the tumor growth behavior in naïve BALB/c mice inoculated subcutaneously (s.c.) with 1 × 10^6^ WTTU, L3TU, or WTTU + L3TU mixture at 1:1 ratio (5 × 10^5^ each) in the flank. While WTTU grew aggressively in 100% of the recipient mice and became measureable by day 7 with an average tumor volume of ~12,000 mm^3^ after 3 weeks, 30% of mice in L3TU group and 20% of mice in the WTTU + L3TU group completely rejected the tumors with the remaining clinically evident tumor sizes being 25- and 6-fold smaller than WTTU, respectively (Figures 1A,B). A 20-fold reduction in the L3TU inoculum (5 × 10^5^ cells) resulted in a 40% tumor-free incidence (Figures 1C,D). As expected, mice that rejected primary L3TU tumors were capable of rejecting subsequent challenge with a lethal dose of WTTU, demonstrating the successful generation of antitumor immune memory (data not shown).

### aCCL3 Augments T Cell Activation by Enhancing Leukocyte Migration to the TDLN

Studies by Gretz and colleagues showed that rCCL3 administered s.c. in the footpad could readily drain though the afferent lymphatic ducts to associate within the high endothelial venules (HEVs) (17). Therefore, we examine how CCL3 produced by L3TU influence cellular traffic to the TDLN during the early phase (≤10 days) of immune priming following tumor inoculation. We examined the TDLN cellularity of CD4^+^ T cell, CD8^+^ T cells, CD19^+^ B cells, CD11c^+^ pAPCs, and CD49b^+^ cells. Both WTTU and L3TU inoculation led to significant increases in CD4^+^, CD8^+^, CD19^+^, CD11c^+^, and CD49b^+^ leukocytes in the TDLN compared to NI group (Figure 2). However, TDLNs draining L3TU showed 2- to 6-fold increases in total leukocyte sub-populations compared to WTTU TDLNs, with the enhancement of leukocyte accumulation reaching statistical significance after 3 days and peaking on days 5 and 7 (Figures 2A–E). These changes were accompanied by gross anatomical differences in LN sizes [Figure S2 in Supplementary Material subcutaneously (s.c.)]. These striking changes were not caused solely by CCL3 alone, as mice receiving direct s.c. injections of rCCL3 showed only transient (<2 days) changes in leukocyte accumulation in TDLN that were similar to PBS controls (Figure 2H). With the exception of CD4^+^ T cells, leukocyte numbers in both WTTU and L3TU TDLN returned to NI levels by day 10. Next, we interrogated whether the significant increases in T cell numbers correlated with changes in the T cell activation status. Mice were injected similarly as above, and both TDLN and contralateral non-draining lymph nodes (NDLNs) were removed on days 1, 3, 5, and 7 for CD69 expression assessment by flow cytometry (Figures 2F,G). In both CD4^+^ and CD8^+^ T cell subsets, we detected greater numbers of CD69^+^ T cells in L3TU TDLN. The number of CD69^+^ T cells began to dissipate after day 5. Interestingly, we also observed a significant and reproducible increase in leukocyte accumulation in the NDLNs on day 5, suggesting a systemic effect of CCL3 on immune cell mobilization and trafficking (Figure S3 in Supplementary Material). However, this effect was transient and quickly dissipated after day 5, and the transient cellularity increase was not accompanied by CD69^+^ T cell activation (Figures S3F,G in Supplementary Material), suggesting a requirement for the presence of tumor cells to sustain the accumulation of CD69^+^ T cell subset in TDLN. Despite differences in absolute cellularity of TDLN and NDLNs between WTTU and L3TU, the overall cellular compositions were similar between the two tumors. Normally, CD4^+^ T cells predominates among LN leukocytes in the naive mouse; however, B cells became the most prominent population proportionally within TDLN 3 days after tumor injections, and the trend continued until after day 7 when the relative composition returned to baseline (Figure S4 in Supplementary Material). The rate of composition reversal by day 10 in TDLN and NDLN between CD4+ T cells and B cells appeared to be more dramatic in L3TU compared to WTTU.

### aCCL3 Bolsters the Intracellular Production of IFNγ+ Cells in the TDLN

**Figure 3:**
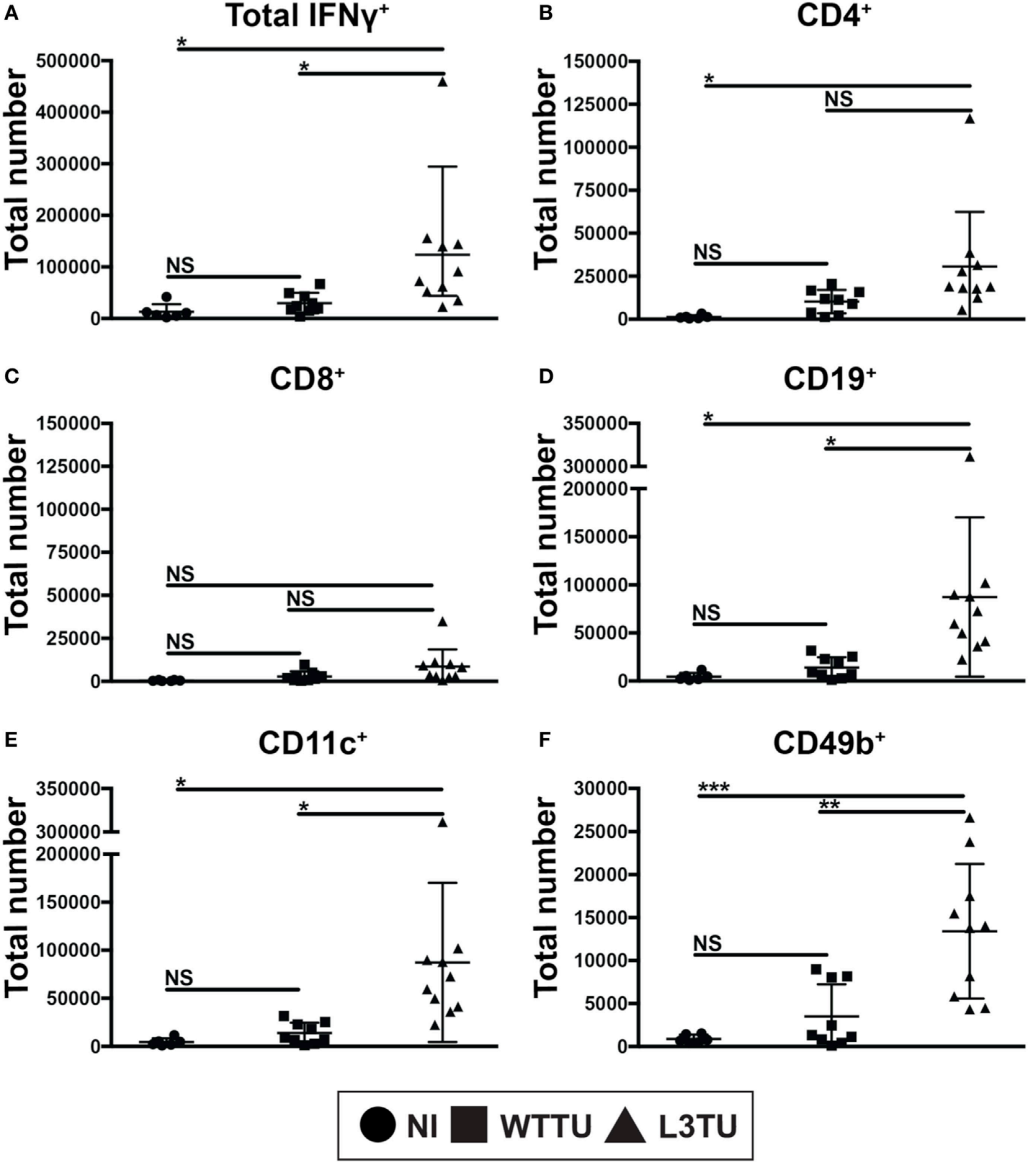
aCCL3 enhances the accumulation of IFNg+ cells in the TDLN. Absolute numbers of **(A)** IFNg+ total immune cells, or IFNg+ immune cells expressing **(B)** CD4, **(C)** CD8, **(D)** CD19, **(E)** CD11c, and **(F)** CD49b in the TDLN on day 5 following tumor inoculations were measured by FACS. *N* = 4–10 mice per group with two repeats. Not significant, ns; *p* > 0.05, *p = 0.01–0.05, **p = 0.001–0.01, ***p = 0.0001–0.001, ****p < 0.0001.

Next, we asked whether the enhanced number of activated CD69+ T cells would correlate with an anti-tumorigenic milieu in TDLN. To address this, following tumor inoculation we measured global cytokine mRNA differences, including TGFβ, TNFα, IL-10 and IFNγ, between WTTU and L3TU groups on day 5, the time point where we observed the greatest differences in cellular accumulation and CD69 positivity. While the average transcript levels of TGFβ, TNFα, and IL-10 in TDLN were similar between L3TU and WTTU, TDLN in L3TU contained a ~2.5-fold greater IFNγ mRNA transcript level as compared to that in WTTU (Figure S5 in Supplementary Material). We then examined intracellular IFNγ content among the cells found within TDLN on day 5. With the exception of CD8+ T cells, all other cell-types analyzed contained significantly elevated numbers of cells expressing intracellular IFNγ in L3TU TDLN (Figure 3). This finding was further confirmed by ELISPOT analysis (data not shown).

### L3TU Enhanced Leukocyte Accumulation Is Dependent on CCL3 but Not NK Cells

**Figure 4:**
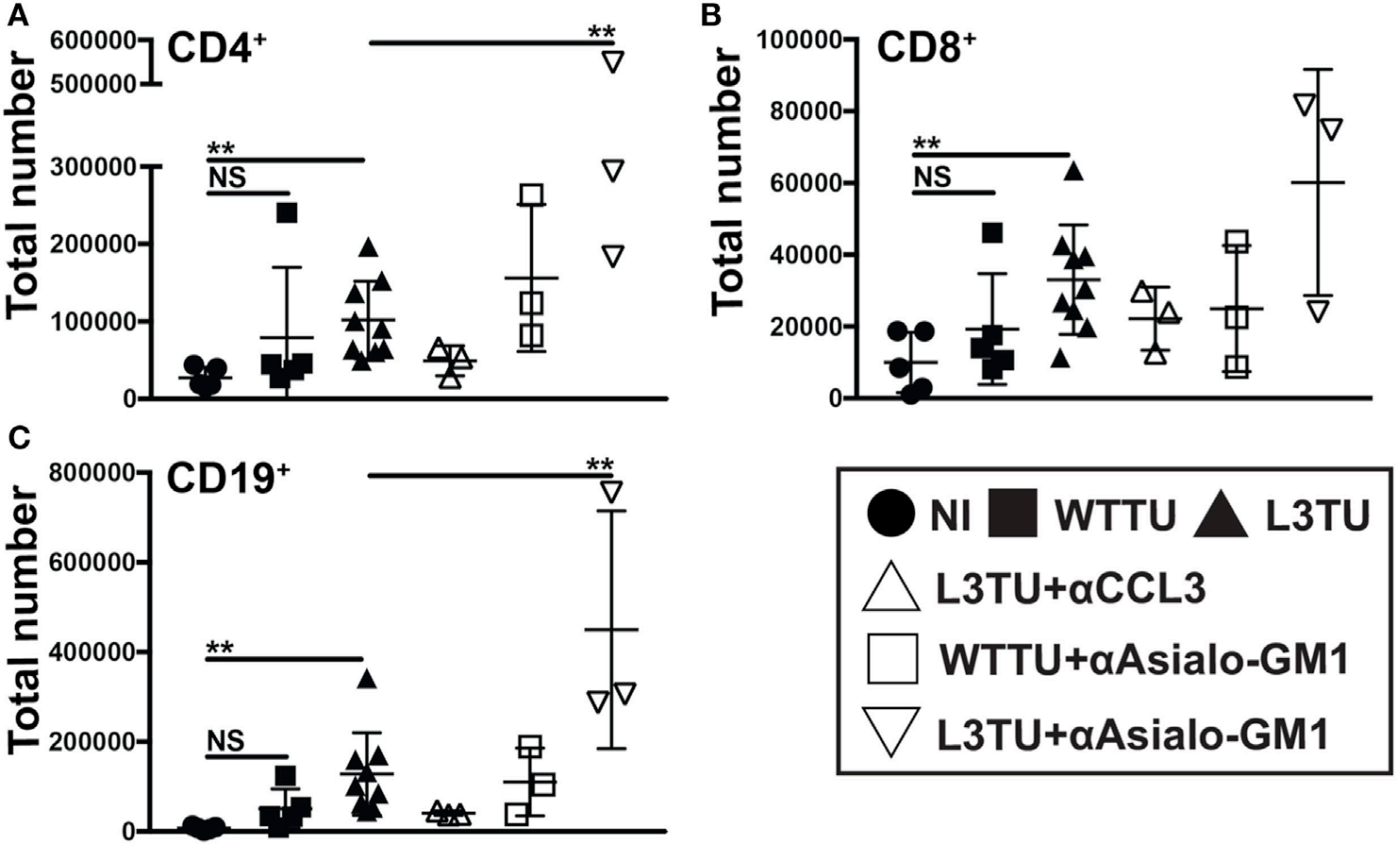
The enhanced accumulation of leukocyte subsets in the L3TU tumor-draining lymph node (TDLN) is negated by blocking CCL3, but enhanced by natural killer (NK) depletion. **(A–C)** Absolute numbers of immune cell subsets in the TDLN 5 days post-tumor injection of mice with and without the anti*-*CCL3 blocking Ab or NK cell (anti-Asialo-GM1) depletion Ab. *N* = 3 mice per cohort. NI (circles), WTTU (squares), and L3TU (triangles) cohorts were conducted concurrently along with the CCL3-blocking and NK depletion cohorts. IgG1a antibody was administered to WTTU, and L3TU cohorts as controls. Not significant (ns), *p* > 0.05; *p = 0.01–0.05; **p = 0.001–0.01; ***p = 0.0001–0.001; ****p < 0.0001.

By day 5, CD49b^+^ cells not only make up the cell-type with the most significant difference between WTTU and L3TU, but they also constituted the most statistically significant IFNγ+ population differences (Figures 2E and 3F). CD49b (VLA-2) is an integrin protein expressed on NK cells, and a subpopulation of NK T (NKT) cells, CD4+ and CD8+ cells (18, 19). NK, NKT, and CD8+ cells have been associated with tumor rejection and IFNγ upregulation after stimulation (20–22). Recently, CD49b+ CD4+ cells were defined as a subpopulation of regulatory T cells that produced IL-10 in response to stimulation rather than IFNγ (23). We hypothesized that the majority of the global IFNγ production within TDLN on day 5 is derived from activated CD49b+ CD3− NK cells, which express CCR5 and could potentially be recruited directly by CCL3 to the TDLN (22). Under homeostatic conditions, NK cells represent a small population (≤1%) in the LN but can accumulate and supply significant IFNγ to drive T cell activation and differentiation upon stimulation (Figure S6 in Supplementary Material) (24). We examined the dependence of cellular accumulation in the L3TU TDLN on CCL3 and NK cells by administering anti-CCL3-neutralizing monoclonal Abs (mAbs) or NK-depleting αAsialo-GM1 antibody prior to footpad inoculation with WTTU or L3TU (Figure S6 in Supplementary Material). We then examined the cellularity of TDLN and NDLN on day 5 as previously described (Figure 4). Blocking CCL3 resulted in diminished cellular accumulation in both TDLN and NDLN (Figure 4 and data not shown). While NK cell depletion over the course of 5 days was associated with a decrease in global IFNγ expression in TDLN (Figure S5 in Supplementary Material), however, counter to expectation, NK cell depletion also potentiated the enhanced cellular accumulation observed with aCCL3 (Figure 4).

### NK Cells, but Not CD103^+^ CD11c^+^ DCs Are Important for Driving the Production of IFNγ-Induced Chemokines CXCL9 and CXCL10 in the TDLN

**Figure 5:**
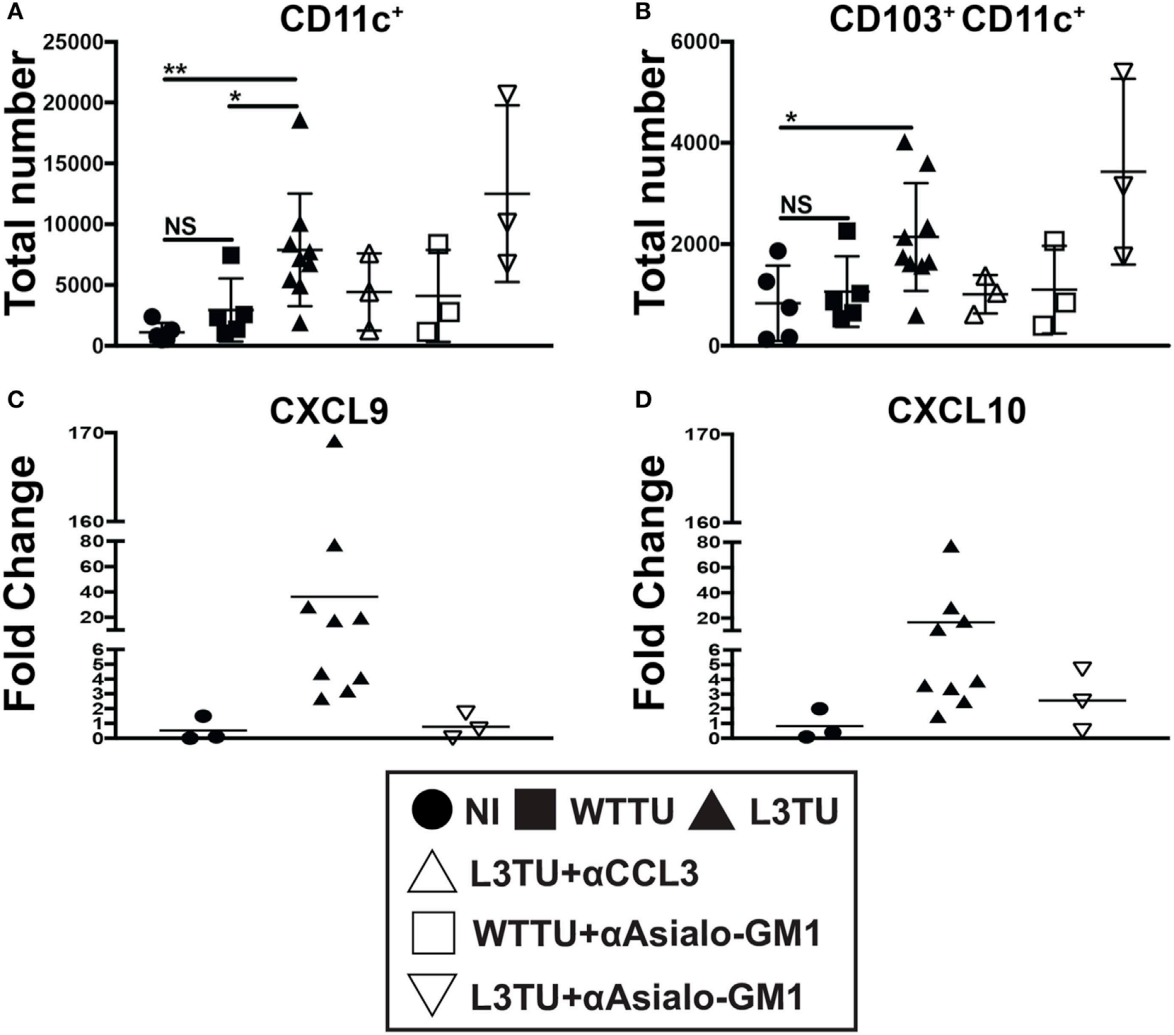
Natural killer (NK) cells, not CD103^+^ CD11c^+^ dendritic cells (DCs), drive IFNγ-induced CXCL9 and CXCL10 production in the L3TU tumor-draining lymph node (TDLN). **(A,B)** Absolute total and CD103^+^ subset of CD11c^+^ cell numbers in the TDLN 5-days post-tumor injections were numerated by FACS. *N* = 3 mice per cohort. non-injected (NI), WTTU, and L3TU injection groups were conducted simultaneously with each depletion experiment and combined into the present graphs. IgG1a antibody was administered to NI, WTTU, and L3TU cohorts as for CCL3 blockade or NK depletion. **(C,D)** Fold change of CXCL9 and CXCL10 mRNA expression in the L3TU TDLN compared to that of WTTU group. Lymph nodes (LNs) from two to three mice in the NI group were pooled for analysis due to low mRNA content. Not significant (ns), *p* > 0.05; *p = 0.01–0.05; **p = 0.001–0.01; ***p = 0.0001–0.001; ****p < 0.0001.

Interferon-gamma expression at primary tumor sites can drive DC maturation and rapid migration to DLN for Ag-presentation (25). IFNγ has also been shown to induce the production of CXCL9 and CXCL10, which can aid in tumor rejection through the recruitment of activated CD8^+^ T cells and IFNγ-secreting Th1 cells to the primary tumor sites (26–28). Recently, studies have shown that CD103^+^ CD11c^+^ DCs, a subpopulation of dermal- and gut-resident pAPCs, respond to tumor-derived CCL4 and are the chief cells that produce CXCL9 and CXCL10 to recruit tumor-infiltrating T cells (29, 30). Indeed, we observed an accumulation of CD103^+^ CD11c^+^ DCs in L3TU TDLN, and such enhanced accumulation was dependent on CCL3, not NK cells or associated IFNγ (Figures 5A,B; Figure S5 in Supplementary Material). However, we show that TDLN NK cells are crucial for CXCL9 and CXCL10 production on day 5 (Figures 5C,D), as NK depletion resulted in a dramatic decrease in the mRNA transcripts of both chemokines.

### Exposure of BMDCs to rCCL3 Enhances Ag-Specific T Cell Proliferation

**Figure 6:**
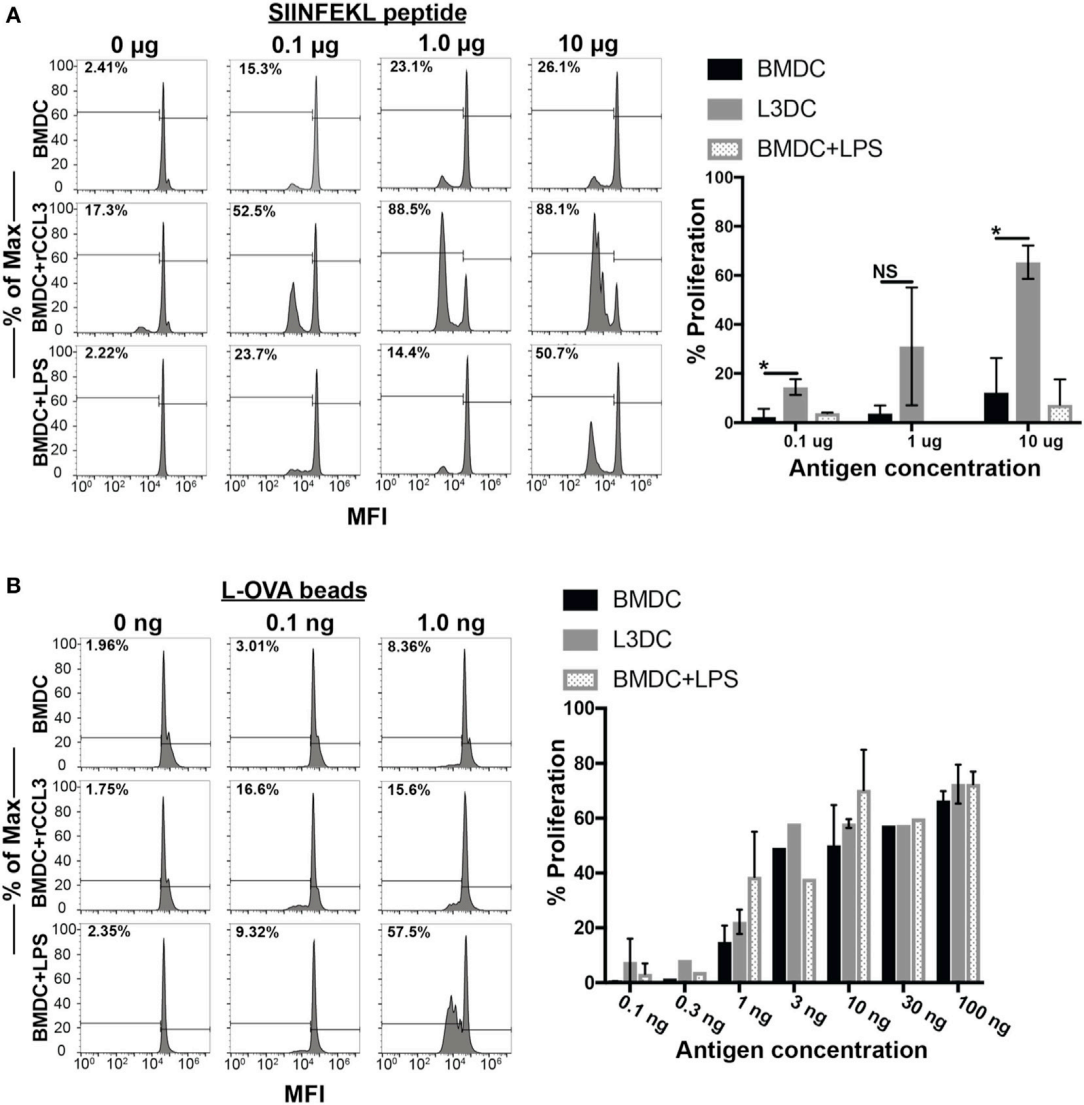
Pretreatment with recombinant CCL3 (rCCL3) enhances the capacity of bone marrow-derived DCs (BMDCs) to drive OT-I proliferation *in vitro*. **(A)** Day 7 BMDCs were cultured for 24 h in the presence or absence of CCL3 (100 ng/ml) or LPS (100 ng/ml), then washed and pulsed with the indicated concentration of SIINFEK peptide, and plated in the presence of CFSE-labeled (1μM) naive OT-I T cells at 1:5 BMDC-to-T cells ratio for 72 h. A total of three independent experiments were performed. **(B)** Day 7 BMDCs were cultured for 24 h with media, 100 ng/ml rCCL3 or LPS (100 ng/ml), then washed and incubated with OVA-latex beads at varying concentrations with CFSE-labeled (1 μM) naïve OT-I T cells at 1:5 BMDC-to-T cells ratio for 72 h. Quantification of FACS data in **(A,B)** are summarized in the bar graphs. Quantification of results in **(A,B)**. Each bar was calculated after subtracting the background CFSE dilution in the absence of added antigen. Each graph is representative of three to four experimental replicates. Not significant (ns), *p* > 0.05; *p = 0.01–0.05; **p = 0.001–0.01; ***p = 0.0001–0.001; ****p < 0.0001.

Finally, we examined the immune-modulatory effects of CCL3 on pAPC function. Several reports have observed the direct modulatory effect of CCL3 on pAPC function. Watanabe and colleagues showed that macrophages pretreated with CCL3 exhibit strengthened adhesion to osteoblasts leading to the formation of pre-osteoclast cells *in vitro*, an important step in the process of bone reabsorption (31). Previously, we showed that blocking CCL3 (and its paralog CCL4) *in vivo* decreased CD8^+^ T cell and DC contacts in the vaccine-draining LN and diminishes the magnitude of the overall CD8^+^ T cell memory pool (5). In two separate studies, Park and colleagues showed that pretreatment of DCs with CCL3 in combination with CCL19 and LPS stimulation led to enhanced OVA-specific CD4^+^ (OT-II) T cell proliferation *in vitro*. To address a potential functional modulatory role of CCL3 on pAPC, we pretreated BMDCs from C57BL/6 mice with rCCL3 for 24 h, then co-cultured them with OT-I T cells for 3 days with and without Ags *in vitro*. CCL3-conditioned BMDC displayed a significantly enhanced capacity to drive OT-I proliferation when pulsed with SIINFEKL peptides at doses of 0.1–10 μg/ml (Figure 6A). Interestingly, CCL3-conditioned BMDC also exhibited enhanced cross-presentation capacity to drive OT-I proliferation when cultured with whole OVA-coupled beads, especially at low Ag doses of 0.1–1 ng/ml (Figure 6B).

## Discussion

Recent studies in melanoma implicate that tumor cells modulate intra-tumoral T cell density in part by regulating inflammatory chemokine productions in the TME *via* a β-catenin-dependent mechanism (29, 32). In particular, Spranger et al. showed melanoma tumor cells that harbor genetic alterations in the β-catenin pathway could upregulate inflammatory chemokine, CCL4, which attracts dermal-resident CD103^+^ DCs. The CD103^+^ DCs elevated CXCL9 and CXCL10 in order to further attract T cells to infiltrate the tumor, and deletion of CCL4 in the melanoma cells abrogated T cell infiltration (29). Although these studies were primarily focused on CCL4, the production of CCL3 was also significantly increased in their tumor system (29). Comparable to these findings, our CT26 colon tumor model does not produce detectable amount of CCL3. Enhancing the production of CCL3 in CT26 by genetic manipulation resulted in significant slowing and ultimate eradication of tumors in a significant fraction of naïve recipient mice. The exact cellular mechanism mediating primary L3TU rejection is the subject of an ongoing parallel study. In the current study, we aimed to distinguish the effect of CCL3 on early antitumor immune priming in the LN from the later adaptive immune responses at the primary tumor site. We did this by assessing CCL3’s effect on global immune cell trafficking and inflammatory changes in the TDLN. Indeed, we observed an increased cellularity in overall CD11c^+^ and CD103^+^ subpopulation of DCs in the TDLN in both WTTU and L3TU as compared to that in NI mice. While there was a slight enhancement of the DC populations in the WTTU TDLN, the magnitude of total and CD103^+^ subset of CD11c^+^ DC accumulation was 2.7-fold and 2-fold less than the accumulation in the L3TU TDLN, respectively (Figures 5A,B). CCL3 was responsible for the increased accumulation of total and CD103^+^ subset of CD11c^+^ cells in L3TU TDLN, as the administration of anti-CCL3 neutralizing antibody abrogated this local LN accumulation (Figures 5A,B). Similar to the observation reported by Spranger et al., we measured a significant increase in the production of CXCL9 and CXCL10 in L3TU TDLN. However, the production of CXCL9 and CXCL10 was disrupted in the L3TU group following NK cell depletion (Figures 5C,D) in conjunction with IFNγ, suggesting that IFNγ plays an important role for promoting CXCL9 and CXCL10 contents in L3TU TDLN. Additional experiments will be required to elucidate this further.

The presence of IFNγ-producing cells in the primary tumor mass is inversely correlated with tumor growth (33). Early presence of IFNγ in the TME favors development of activated DCs and T cells with an inflammatory phenotype. Both CCL3 and IFNγ are implicated in endowing DCs the ability to polarize toward the establishment of type-1 inflammatory responses in T cells, CD8^+^ T cell proliferation, and immune memory generation (3, 26, 34–37). Interestingly, while a greater number of CD8^+^ T cells expressed CD69 in the L3TU groups on day 5, IFNγ production by these CD8^+^ T cells was not significantly different than those found in WTTU TDLN or NI LN, suggesting that the full activation and effector function acquisition of CD8^+^ T cells in L3TU TDLN occurred at a later time point beyond day 5 (Figures 2G and 3C). An unexpected finding was that, compared the WTTU TDLN and NI LN, only L3TU TDLN contained significantly elevated IFNγ^+^ B cells on day 5. A study by Bao et al. showed that a subpopulation of innate secondary-lymphoid-resident B cells could drive the activation of macrophages through IFNγ (38). These IFNγ-expressing innate B cells were shown to accumulate in secondary lymphoid organs as early as 3 days following bacterial or LPS challenge, similar to our current observation with aCCL3 (38). These IFNγ^+^ B cells were the second most abundant early source of IFNγ^+^ cells in the L3TU TDLN (Figure 3D) (39), suggesting that CCL3 may contribute directly or indirectly to the development or accumulation of this particular B cell subset. The significance of these IFNγ^+^ B cells in the antitumor responses remains to be fully explored.

In the present study, CD49b^+^ NK cells represented the most significant source of IFNγ in the L3TU on day 5, as depletion of this immune subset diminished the global IFNγ production in the TDLN (Figure S5 in Supplementary Material). Previous reports showed that the major source of CXCL9 and CXCL10 within the LN comes from LN-endothelial cells and DCs, respectively (40). NK cells are an important source of early IFNγ for LN DCs, and they are recruited to the DLNs by mature DCs in a CCR7-independent and CXCR3-dependent manner though DC production of CXCL10 (26). Therefore, it was not surprising to observe a significant decrease in IFNγ production following NK depletion. We expected that the presence of IFNγ^+^ NK cells might synergistically increase the production of CXCL9 and CXCL10 in the TDLN. Indeed, depleting NK cells as a major provider of IFNγ resulted in a global decrease of CXCL9 and CXCL10, despite the apparent CCL3-driven increases in CD103^+^ CD11c^+^ DCs to the TDLN (Figure 5). A surprising observation in our study was the observed additional enrichment in the overall cellular recruitment in the L3TU group after NK depletion (Figures 4 and 5). However, two separate studies have reported similar phenomenon with neutrophil recruitment to the DLNs of immunized mice (41, 42). CCL3 has also been shown to contribute to tissue-homing of neutrophils during microbial infections (43). In addition, CCL3 expression can be suppressed by IFNγ to auto-regulate inflammatory responses in tissues (44). In our system where CCL3 is continuously elevated in L3TU (Figure S1C in Supplementary Material), it is plausible that the depletion of NK cells—the major source of IFNγ—may further enhance the recruitment capacity of CCL3 in L3TU TDLN by removing the IFNγ-mediated suppressive mechanism on local LN immune cell populations. The exact mechanisms and the associated immune effector function in NK-depleted L3TU TDLN remains to be explored.

We show that s.c. administration of PBS alone can briefly enhance leukocyte migration patterns temporarily and locally through transient increases in the interstitial pressure in the DLN. Ultimately, however, the DLN responds quickly by returning to homeostasis within 24–48 h (Figure 2). The s.c. administration of rCCL3 alone was also not enough to induce a sustained increase in leukocyte traffic to the LN (Figure 2). This observation agrees with published literature showing that CCL3 could drive T cell emigration from peripheral blood to tissues only under the influence of immunogens such as dinitrofluorobenzene (45). Our data suggest that factors derived from tumor cells are also critical for CCL3-induced TDLN accumulation of leukocytes.

While enhancing T cell recruitment can increase the efficiency of their scanning of potential cognate Ag on DCs, delivery of relevant Ag to TDLN is just as vital for eliciting robust T cell responses. Cytokines such as CCL3 can begin to affect cellular responses within the TDLN early in this process before metastasizing tumors or skin migratory DCs could directly influence the immune responses (39). The current dogma dictates that DC migration to the DLN from tissues occurs in a CCR7-dependent manner. Interestingly, however, we show that DC migration to the TDLN is enhanced by aCCL3 (Figure 5) (29, 46). Furthermore, aCCL3 modulates DC’s functional ability to induce T cell proliferation *in vitro*. Jaehyung and colleagues showed that CCL3-exposed DCs enhance OT-II proliferative capabilities in an Ag-specific manner, but only after co-stimulation with CCL19 and LPS (47), which maintain exposed DCs in a semi-mature state to allow for greater Ag-uptake and subsequent loading onto MHC molecules for presentation to T cells. Yanagawa and Onoe observed that short-term (1 h) exposure of DCs to CCL3 could directly activate the endocytotic pathway in immature DCs, suggesting that the initial uptake of Ag by DCs could be enhanced in the short term (48). However, we failed to detect any augmentation of MHC, CD40, CD80, or CD86 expression on the surface of DCs after 24 h of exposure to CCL3 alone (data not shown) as an explanation for the observed enhanced T cell proliferation. Furthermore, we also did not detect any significant changes in intracellular fluorescence intensity when we exposed 24-h CCL3-cultured DCs to fluorescently labeled Latex-OVA beads to detect differences in Ag uptake (data not shown). While we cannot account for the enhancement in Ag-uptake with short-term exposure of DCs to CCL3, our data suggest that prolonged exposure of DCs to CCL may facilitate enhanced processing rather than uptake of Ag.

Taken together, our data implicate a direct immune-modulating effect of CCL3 in the TDLN through the accumulation of IFNγ^+^ NK cells and CD103^+^ DCs, enhanced production of CXCL9 and CXCL10, improved Ag-presentation and stimulation capacity of DCs, and improved T and B-lymphocytes activation. Our current data further support the exploration of CCL3 as an adjuvant for enhancing antitumor immune response.

## Ethics Statement

This study was carried out in accordance with the recommendations of National Institutes of Health institutional guidelines. The protocol was approved by the Case Western Reserve University Institutional Animal Care and Use Committee (No. 2012-0126 and 2015-0118).

## Author Contributions

FA conducted, designed, or took part in the implementation of all experiments, analysis, and interpretation of the analysis within this paper. FA also contributed as the writer for this paper. PR help collect samples and conducted all the qPCR experiments as well as helped with the final analysis. DA conducted the T cell proliferation assays. AT helped with the tumor growth and antibody depletion studies. JN helped designed the L3TU, GFP-WTTU, and GFP-L3TU constructs for these experiments. SE helped with the IFNγ ELISPOT design and experiments for the proof of concept experiments. JM and CT help with designing the tumor constructs (those used and not used) as well as the experiment and collection of preliminary data for the funding of this project. AH is the principle investigator and senior author for this project.

## Conflict of Interest Statement

The authors declare that the research was conducted in the absence of any commercial or financial relationships that could be construed as a potential conflict of interest.

## Abbreviations

LN, lymph node; NDLNs, non-draining lymph nodes; TDLNs; tumor-draining lymph nodes; WTTU, wild-type CT26 tumor; L3TU, CCL3-secreting CT26 colon tumor; APC, antigen-presenting cell; DCs, dendritic cells; TME; tumor microenvironment; aCCL3, autologous tumor-derived CCL3; rCCL3, recombinant CCL3; pAPCs, professional Ag-presenting cells; NK, natural killer cells; NI, non-injected; HEVs, high endothelial venules; NKT, NK T cells; BMDCs, bone marrow-derived DCs; Ag, antigen; s.c., subcutaneous.
