# Supplementary material for: CCL3 Enhances Antitumor Immune Priming in the Lymph Node via IFNγ with Dependency on Natural Killer Cells
Source: Front Immunol. 2017 Oct 23;8:1390. doi: 10.3389/fimmu.2017.01390 (PMC5660298; doi:10.3389/fimmu.2017.01390)
Supplement: Supplementary file 1 [file Data_Sheet_1.DOCX]

Supplementary Material

CCL3 enhances antitumor immune priming in the lymph node via IFN𝛾 with dependency on NK cells

**Frederick Allen, Peter Rauhe, David Askew, Alexander A. Tong, Joseph Nthale, Saada Eid, Jay Myers, Caryn Tong, Alex Y. Huang***

*** Correspondence:** Alex Y. Huang: alex.y.huang@case.edu

**6 Supplementary Figures:**


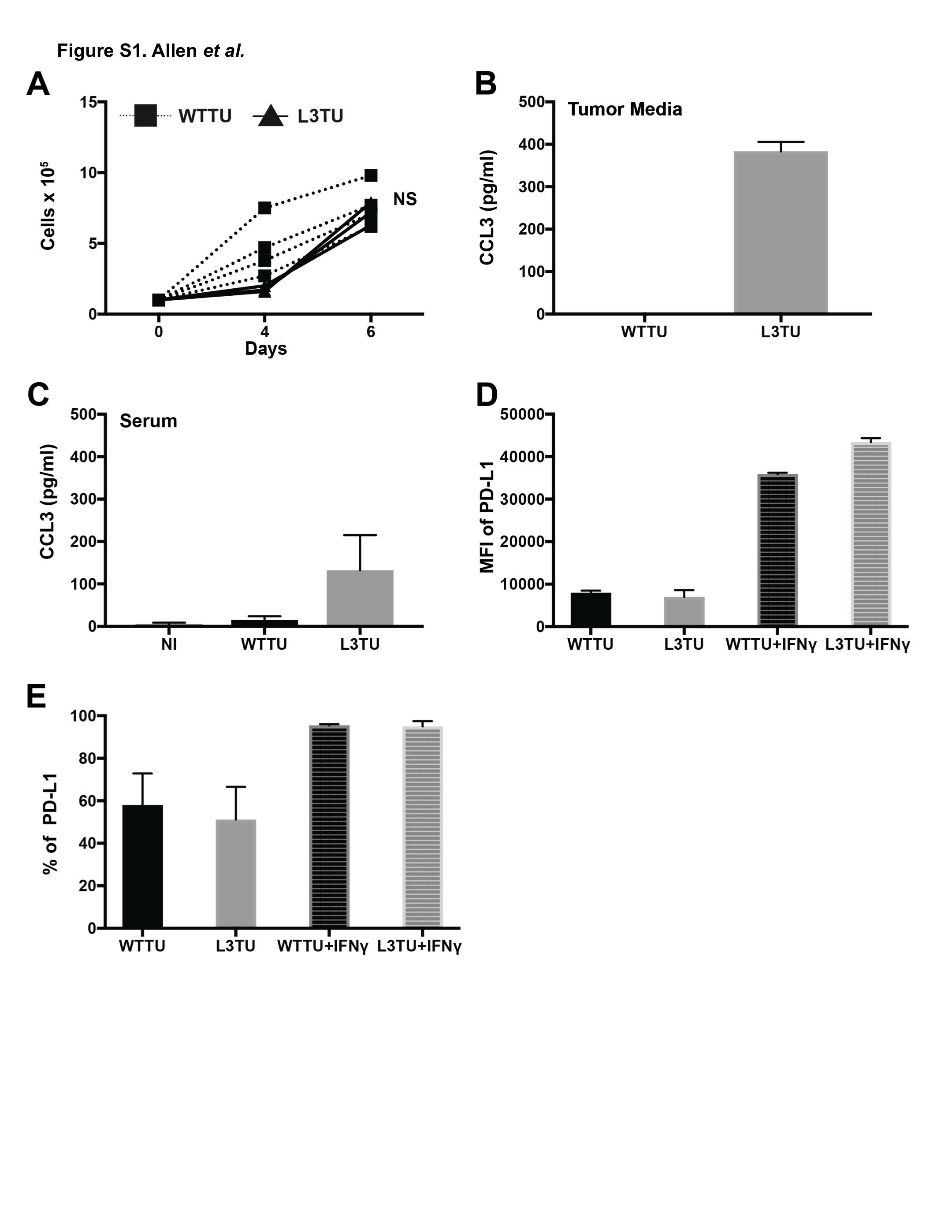


**Supplementary Figure S1. WTTU and L3TU exhibit similar proliferation and PD-L1 expression profiles *in vitro.*** A) *In vitro* proliferation assay measuring the growth kinetics between WTTU and L3TU. B) ELISA of CCL3 proteins secreted from tumor cultures *in vitro*. C) CCL3 ELISA of serum from control or mice 7 days after inoculation with WTTU or L3TU. D) Mean fluorescence intensity (MFI) of PD-L1 expressed on tumor cells before and after 24 hour IFN𝛾 stimulation *in vitro*. E) Percent of WTTU and L3TU cells that express PD-L1 before and after 24 hour IFN𝛾-stimulation *in vitro*. Not significant (ns), p > 0.05; *, p = 0.01 to 0.05; **, p = 0.001 to 0.01; ***, p = 0.0001 to 0.001; ****, p < 0.0001.

**
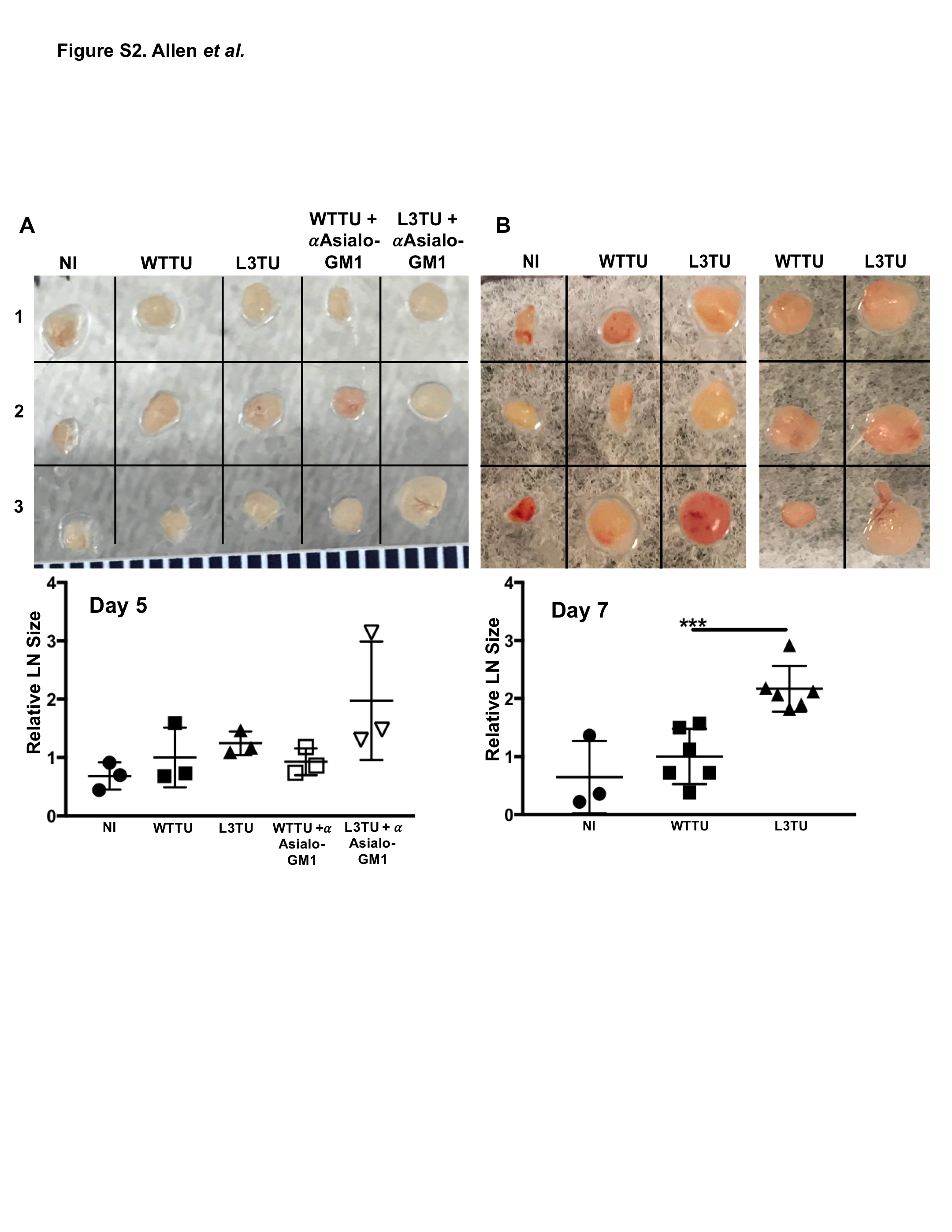
**

**Supplementary Figure S2. Gross anatomical LN images show enlarged TDLN in the L3TU group compared to the WTTU group.** Gross photographs were taken of the popliteal LN of NI, TDLN and NDLN of WTTU, L3TU, WTTU+𝛼Asialo-GM1, and L3TU+𝛼Asialo-GM1 cohorts 5 days (A) or 7 days (B) following tumor inoculations. Measurements of LN sizes are depicted below the photographs. Not significant (ns), p > 0.05; *, p = 0.01 to 0.05; **, p = 0.001 to 0.01; ***, p = 0.0001 to 0.001; ****, p < 0.0001.


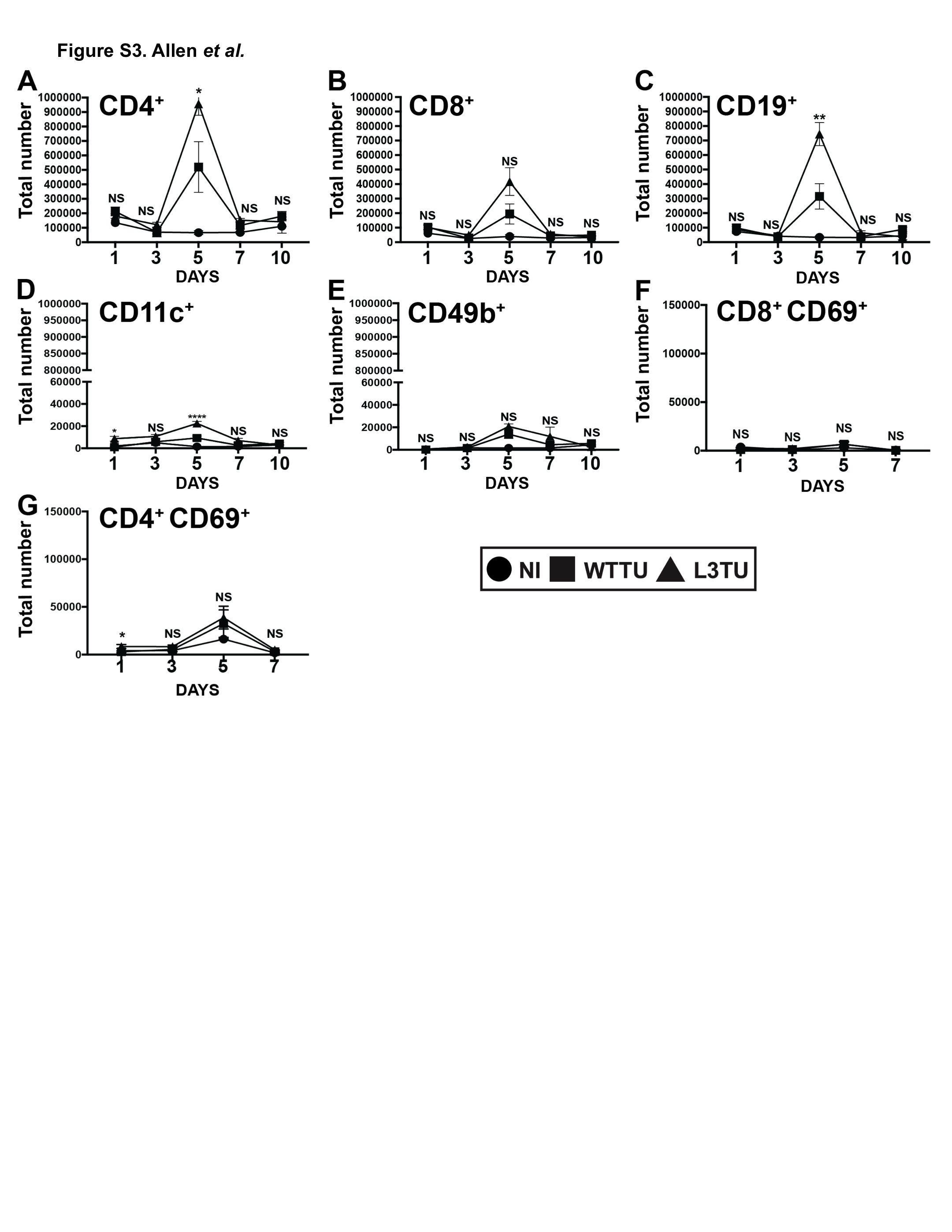


**Supplementary Figure S3. Leukocytes transiently accumulate in NDLNs following L3TU inoculation.** A-E) Cellular accumulations in the NDLNs are shown for the 10 days following tumor inoculation. F-G) Accumulations of CD69^+^ T cell subsets were enumerated using FACS over 7 days following tumor inoculation. N = 2 to 7 mice per cohort per day. Not significant (ns), p > 0.05; *, p = 0.01 to 0.05; **, p = 0.001 to 0.01; ***, p = 0.0001 to 0.001; ****, p < 0.0001.

**
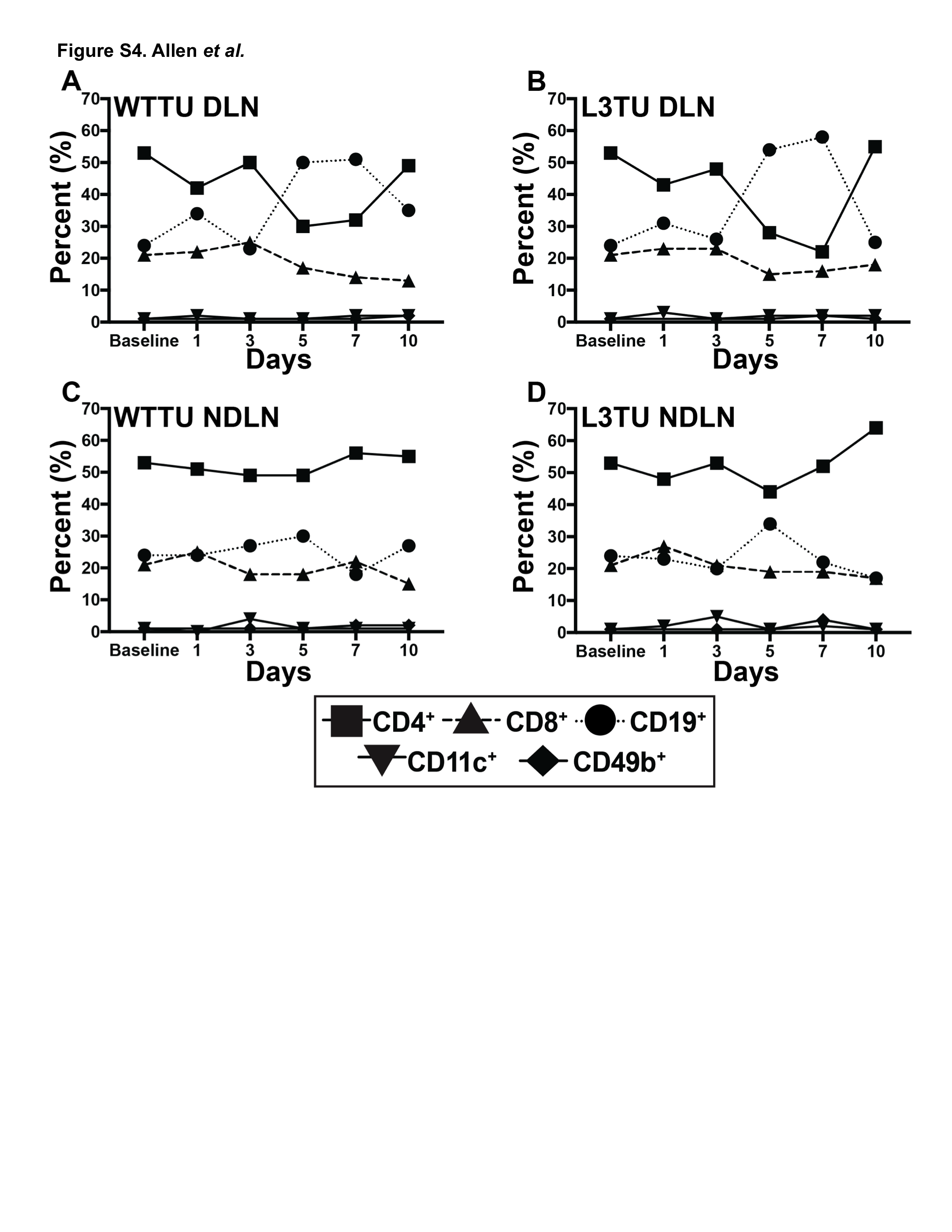
**

**Supplementary Figure S4. Comparison of the leukocyte compositions in WTTU and L3TU TDLN cohorts.** Percent composition of various immune subsets in DLN (A, B) and NDLN (C, D) were enumerated using FACS during the first 10 days following WTTU (A, C) and L3TU (B, D) inoculation. Calculations were based on experiments in Figure 2. Percent composition for each day was calculated using the ratio between the average absolute cell numbers of CD4^+^, CD8^+^, CD19^+^, CD11c^+^ or CD49b^+^ cells and the total cellularity and multiplied by 100. The baseline represents the average absolute number values calculated from NI mice for each cell subset.


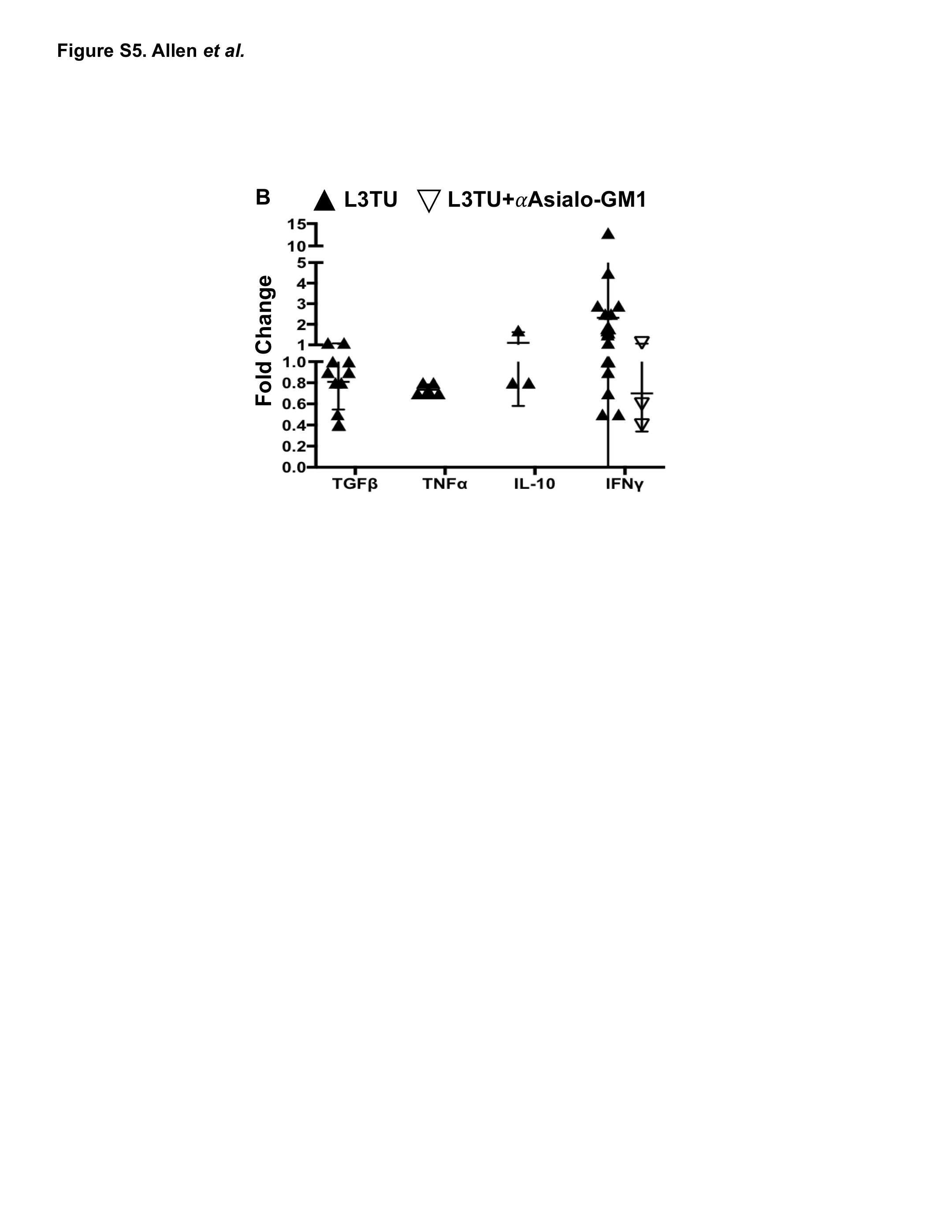


**Supplementary Figure S5. Quantitative cytokine mRNA profiles in the L3TU TDLN**. A) Various cytokine mRNA content within L3TU TDLN was analyzed 5 days following tumor inoculation. The graph shows fold changes between each L3TU replicate and that of WTTU. Each individual symbol represents a biological replicate. TGF𝛽 analysis was repeated 3 times; TNF𝛼, 2 times; IL-10, once; IFN𝛾 6 times; and IFN𝛾 with 𝛼Asialo-GM1, once.


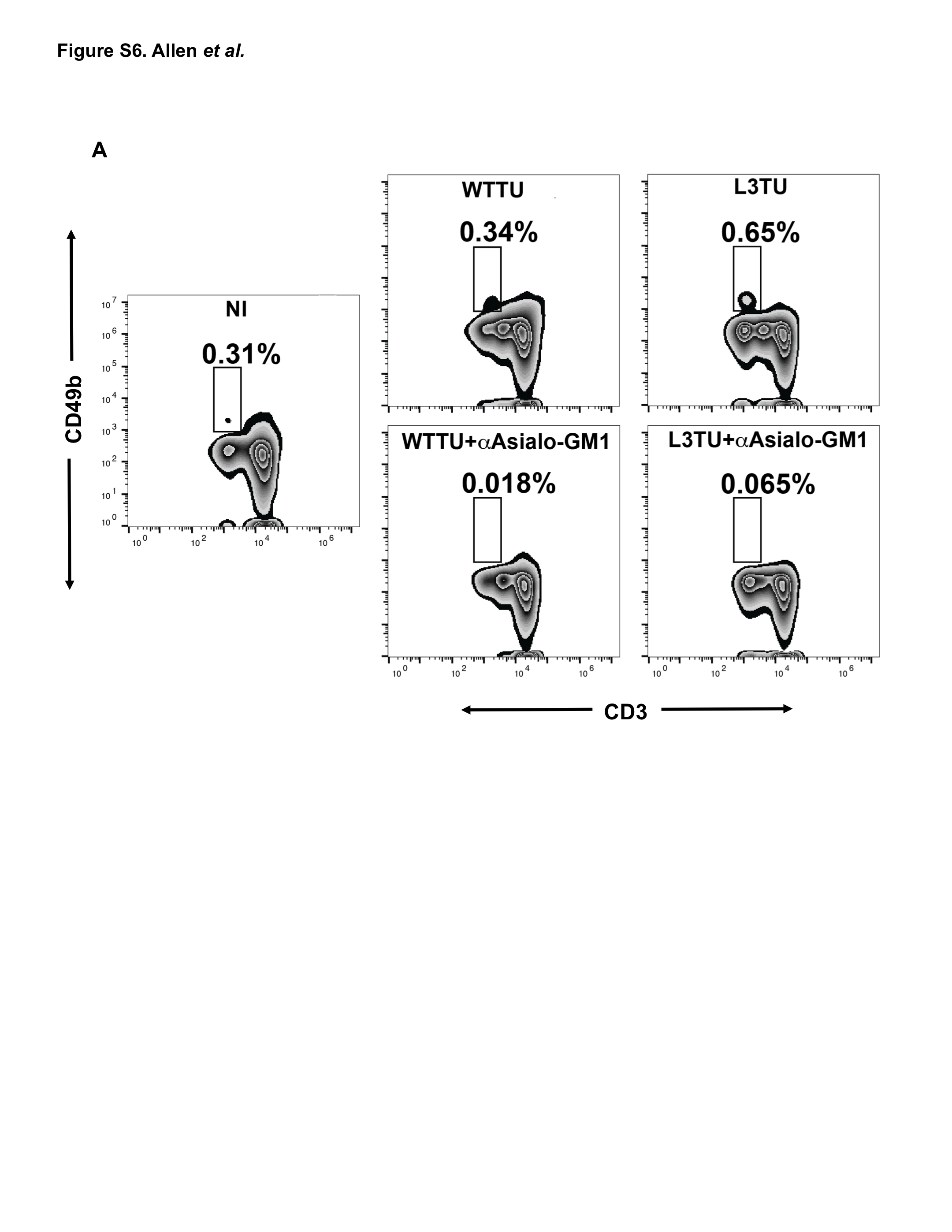


**Supplementary Figure S6. Efficiency of NK cell depletion in the WTTU and L3TU TDLN cohorts.** Representative FACS analysis of NI, WTTU, or L3TU TDLNs 5 days following tumor inoculation. N = 3 mice per cohort.
